# Changes of Vegetation Distribution in the East Dongting Lake After the Operation of the Three Gorges Dam, China

**DOI:** 10.3389/fpls.2018.00582

**Published:** 2018-05-01

**Authors:** Jia-Yu Hu, Yong-Hong Xie, Yue Tang, Feng Li, Ye-Ai Zou

**Affiliations:** ^1^Key Laboratory of Agro-Ecological Processes in Subtropical Region, Institute of Subtropical Agriculture, Chinese Academy of Sciences, Changsha, China; ^2^Dongting Lake Station for Wetland Ecosystem Research, Institute of Subtropical Agriculture, Chinese Academy of Sciences, Changsha, China; ^3^University of Chinese Academy of Sciences, Beijing, China

**Keywords:** vegetation distribution, water regime, Three Gorges Dam, East Dongting Lake wetland, submergence duration, lowest distribution elevation

## Abstract

Water regime is regarded as the primary factor influencing the vegetation distribution in natural wetland ecosystems. However, the effect of water regime change induced by large-scale hydraulic engineering on vegetation distribution is still unclear. In this study, multi-temporal TM/ETM+/OLI images and hydrological data from 1995 to 2015 were used to elucidate how the change in water regime influenced the vegetation distribution in the East Dongting Lake (EDTL), especially after the operation of the Three Gorges Dam (TGD) in 2003. Using unsupervised and supervised classification methods, three types of land cover were identified in the study area: Water and Mudflat, Grass, and Reed and Forest. Results showed that the total vegetation area in EDTL increased by approximately 78 km^2^ during 1995–2015. The areas of Reed and Forest and Grass exhibited a contrasting trend, dramatic increase in Reed and Forest but sharp decrease in Grass, particularly after the operation of TGD. The lowest distribution elevations of Grass and Reed and Forest decreased by 0.61 and 0.52 m, respectively. As a result of water level variation, submergence duration increased at 20–21 m and 28 m elevations (1–13 days), but significantly decreased at 22–27 m and 29–30 m elevations (-3 to -31 days). The submergence duration of Grass and Reed and Forest was 246 and 177 days, respectively. This study indicated that wetland vegetation pattern significantly changed after the operation of TGD, mainly as a result of changes in submergence condition. Submergence duration might be an effective indicator to predict the shift of vegetation distribution in EDTL, and which could provide scientific guidance for vegetation restoration and wetland management in this lake.

## Introduction

Wetland is a crucial component of the earth’s landscape and is considered as one of the most diverse and productive ecosystems on the planet (Kingsford, 2000; Rosenberg et al., 2000; Tockner and Stanford, 2002). They can store large quantities of water in regular wet season and also provide a buffer zone and a place of energy exchange for the development of flora and fauna in dry season (Sedell et al., 1990; Semlitsch and Bodie, 2003; Zedler and Kercher, 2005). However, wetland is a sensitive ecosystem. Variations in water regime, such as the frequency, duration, magnitude, and timing of flooding, can transform some key environmental factors for the development of downstream wetlands (Poff et al., 1997). These changes directly or indirectly impact wetland structure and community dynamics (Miller and Zedler, 2003; Zedler and Kercher, 2005; Fraser and Miletti, 2008; Todd et al., 2010), sometimes even resulted in the disappearance of flood-dependent vegetation (Auble et al., 1994; Taylor et al., 1996; Arthington and Pusey, 2003).

Water level is considered as a major factor influencing the development of wetlands and an intuitive indicator to assess the changes of vegetation distribution, since water level fluctuation strongly affects the growth and survival of vegetation (Casanova and Brock, 2000; Johansson and Nilsson, 2002; Coops et al., 2003). Zonation is a general response of wetland vegetation to regular water level fluctuation. In seasonal temporary wetlands, woody vegetation usually distributes at a higher elevation where there is less water level fluctuation and even no submergence. On the other hand, the herbaceous plant usually distributes at a lower elevation where there is usually under constant and regular submergence. In some particular regions, vegetation will shift to lowland types within a few years when the water level keeps at higher level. In other regions, the community types will be easily replaced by the perennial types with clonal reproduction when the water level keeps at lower water level (Chapin and Paige, 2013). Therefore, the pattern of vegetation distribution appears to be unclear when water level is variable.

**FIGURE 1:**
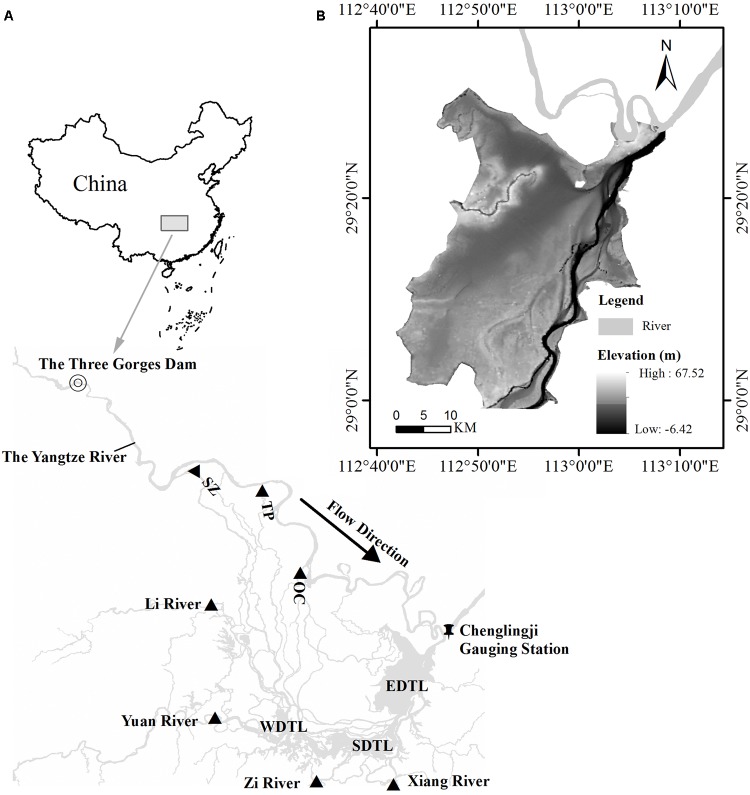
Location of the East Dongting Lake. **(A)** Relationship between the TGD and the Dongting Lake. SZ–Songzi Channel; TP-Taiping Channel; OC-Ouchi Channel, **(B)** Digital elevation model of East Dongting Lake.

Due to interference of human activities, such as hydroelectricity generation and flood control (e.g., dam building and river management) over 60% of the global river systems, the downstream wetlands have been affected by altered stream flows (Revenga et al., 2000). Dongting Lake wetland is a typical example, which receives water inflow from three channels (Songzi, Taiping, and Ouchi) directly connected to the Yangtze River and four rivers (Xiang, Zi, Yuan, and Li) in Hunan Province (**Figure 1**). This lake and its wetland have been threatened by unpredictable and dynamic changes in water regime, particularly after the operation of the Three Gorges Dam (TGD) in 2003, the largest hydrological project in the world. Several studies have confirmed that the TGD has changed the water regime of downstream (Zhang et al., 2012; Gao et al., 2013; Wang et al., 2013). Specifically, the TGD directly influenced the flooding patterns of wetlands, local vegetation and habitat of migratory birds in both Dongting Lake and Poyang Lake (Han et al., 2015; Mei et al., 2015; Feng et al., 2016; Wu and Liu, 2017). Even though these studies have elucidated the changes of eco-hydrological environment in the downstream lakes and wetlands after the operation of TGD, the relationship between vegetation distribution and water regime remains largely unclear, particularly in the Dongting Lake wetland.

In recent years, remote sensing and RS/GIS techniques provide the possibility to investigate vegetation distribution over large scales (Murkin et al., 1997; Munyati, 2000; Frazier et al., 2003; Accad and Neil, 2006; Rebelo et al., 2009). This study aimed to use these techniques to investigate the change of vegetation distribution in the East Dongting Lake (EDTL) in response to the water regime change. The specific objectives of the present study are as follows: (1) to characterize the change of vegetation distribution in the EDTL during 1995–2015, and (2) to explore the relationship between the dominant vegetation distribution and changes of water regime.

## Materials and Methods

### Study Area

Dongting Lake (28°44′ and 29°35′N, 111°53′ and 113°05′E), the second largest freshwater lake (2,625 km^2^) in China, is located in the northeast of Hunan Province (**Figure 1A**). This lake is one of the only two river-connected lakes in the Yangtze River Basin. Therefore, its water level is strongly influenced by the Yangtze River, especially after the operation of TGD (Sun et al., 2012). It consists of three sub-lakes: the East, West, and South Dongting Lakes and all of them are international important wetlands (Ramsar Sites).

The EDTL, covering approximately 1,321 km^2^, accounts for nearly half of Dongting Lake’s area. This wetland is characterized by a distinct plant zonation pattern along elevation gradients. For instance, the dominant plant communities *Polygonum hydropiper*, *Carex brevicuspis*, *Miscanthus sacchariflorus*, *Phragmites australis*, and *Populus nigra* are usually distributed along with increasing elevations (Li et al., 2013). The vegetation, especially grass (mostly Carex spp.), provides important habitat for winter migratory waterbirds (Yuan et al., 2014). The lake is usually inundated during the wet season (May–October) and exposed to air during the dry season (November–April). The annual fluctuation of water level is up to 10–12 m, the maximum and minimum water levels occur in July–August and January–February, respectively.

### Datasets and Processing

In this study, 12 time-series of dual-season Landsat TM/ETM+/OLI images of EDTL were chosen (**Table 1**). These images, obtained from the United States Geological Survey (USGS) archives, included winter and pre-winter scenes for each period. Winter images were used because vegetation characteristics are relatively stable and are easily identified in the winter. Meanwhile, pre-winter images were used to eliminate the disturbances of reed harvest in the winter. Winter images were chosen when the daily water level was < 23 m, and the potential vegetation area can be completely exposed to the air, based on the investigation of Dongting Lake Station for Wetland Ecosystem Research, CAS.

**Table 1:** The list of Landsat images.

Season	Date	Landsat images	Water level (m)
Winter	1995/12/05	Landsat5 TM	21.49
Pre-winter	1996/10/04	Landsat5 TM	26.24
Winter	2001/01/11	Landsat7 ETM+	21.86
Pre-winter	2001/10/18	Landsat5 TM	26.40
Winter	2003/01/17	Landsat7 ETM+	22.01
Pre-winter	2003/10/16	Landsat7 ETM+	26.63
Winter	2006/01/09	Landsat7 ETM+	20.79
Pre-winter	2004/10/02	Landsat7 ETM+	27.40
Winter	2010/12/22	Landsat7 ETM+	22.88
Pre-winter	2010/10/03	Landsat7 ETM+	27.32
Winter	2015/03/31	Landsat8 OLI	22.87
Pre-winter	2014/10/22	Landsat8 OLI	24.10

Because some pixels are missing in the ETM+ images after the 31 May 2003 due to scan line corrector (SLC) failure, these images were corrected using the gap-fill algorithm proposed by Scaramuzza et al. (2004). The imagery preparation and classification were performed using ENVI 4.8, while the map production and accuracy assessment were done using ArcMap 10.2.

The field data on vegetation type were acquired from Dongting Lake Station for Wetland Ecosystem Research, CAS in 2014 (a total of 231 data). These data were used for the validation of classification results. The Digital Elevation Model (DEM, 1:10000) of Dongting Lake used in this study (**Figure 1B**), was provided by the Changjiang Water Resource Commission. Based on this DEM, the elevation data (integer) of EDTL was extracted and then converted to WGS84 coordinate system.

Data on daily water level at 8:00 AM at Chenglingji Hydrological Gauging Station during 1990–2015 were also provided by the Changjiang Water Resource Commission. These data are widely used to assess water regime in the EDTL (Hu et al., 2015; Xie et al., 2015; Yuan et al., 2015).

### Image Classification

Generally, only one image at a given time was usually applied for the Remote Sensing image interpretation. However, the conventional classification methods may not be suitable for some wetlands, especially for the seasonal wetlands with high intensity of human disturbance. Therefore, dual images were combined into one image to generate a complete wetland map, and unsupervised and supervised classification methods were conducted using ISODATA (Iterative Self-Organizing Data Analysis Technique) and DTC (Decision Tree Classification) techniques according to the previous study (Xie et al., 2015). The winter images were classified into water, mudflat, and vegetation using ISODATA algorithm with five iterations and 0.95 threshold. After that, DTC algorithm was applied to extract Reed and Forest from pre-winter images using Band 4 and Band 7 (Xie et al., 2015). Finally, three types of land cover were differentiated for each map: Water and Mudflat, Grass, and Reed and Forest. The subtypes of Grass mainly included *C. brevicuspis* and *P. hydropiper*, while Reed and Forest included *M. sacchariflorus*, *P. australis, Salix babylonica* and *P. nigra.*

### Accuracy Assessment

Based on the field data, the accuracy of classification results is evaluated using the ENVI 4.8 confusion matrix tool. We randomly chose 1000 pixels in the study area and compared them with the 2014 field data. Kappa Index and overall accuracy were then calculated to assess the accuracy of vegetation classification.

### Area and Elevation of Vegetation Distribution

Six-period land cover maps were produced after classification. The areas of the different land cover types (Water and Mudflat, Grass, and Reed and Forest) were calculated by multiplying the pixel number with the spatial resolution (30^∗^30 m). Total vegetation area included the areas of Grass and Reed and Forest.

Furthermore, the land-cover maps were overlaid with elevation data to analyze the elevation distribution of each land cover type. Bezier curve was used to fit the distribution and detect the intersection between land cover types according to the method of Xie et al. (2015). The intersection elevation of two curves was then defined as the distribution edge of two land cover types.

### Calculation of Monthly Water Level and Submergence Duration

Average monthly water level was calculated using the daily water level at Chenglingji Station. The submergence duration was calculated using the daily water level data at 20–30 m elevations with 1 m interval. When the water level was higher than the defined elevation, the wetland was considered as submergence. In order to compare the change of submergence duration and average monthly water level before and after the operation of TGD, daily water level data were divided into two periods: 1990–2002 and 2003–2014. The change of submergence duration was calculated by the submergence days before and after the operation of TGD at a given elevation.

Considering the lag time in the change of vegetation distribution induced by the changed water regime, the water level data of 1–5 years before a reference year were used to calculate submergence duration. For example, taking 1995 as the reference year, we calculated the average submergence duration for each of the following year/year range: 1994, 1993–1994, 1992–1994, 1991–1994, and 1990–1994. Pearson correlation analysis was used to determine the best lag time, then we calculated the submergence duration for each vegetation type using the average daily water level during 1990–2015. Finally, a linear regression model and ANOVA were used to identify the change trend in water level, corresponding to the submergence duration of each vegetation type during 1995–2015.

## Results

### Assessment of Classification Accuracy

The overall accuracy and Kappa coefficient were 92.2% and 0.875, respectively. The products accuracy and user accuracy were highest in Water and Mudflat (94.69 and 98.57%, respectively), intermediate in Reed and Forest (94.66 and 89.21%, respectively), and lowest in Grass (83.91 and 82.48%, respectively). These results confirmed that the classification method was valid.

### Changes in Land Cover Area

**FIGURE 2:**
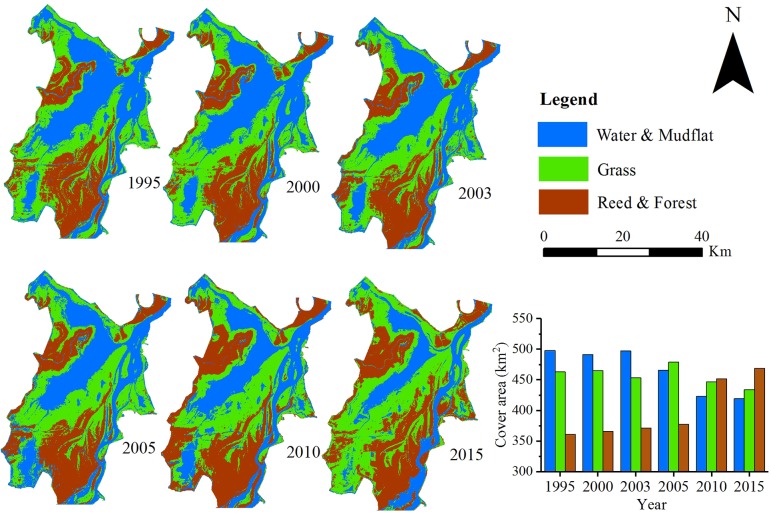
Land cover change in the East Dongting Lake during 1995–2015.

The area of three types of land cover showed different pattern during 1995–2015 (**Figure 2**). The area of Water and Mudflat continually decreased from 497.7 km^2^ in 1995 to 419.4 km^2^ in 2015. The Grass area also decreased from 463.0 to 433.5 km^2^ during 1995–2015. Specially, the Grass area actually experienced a marked increase from 2003 to 2005, after which it began to decrease. On the contrary, the area of Reed and Forest increased from 361.1 to 468.8 km^2^ during the same period. Meanwhile, total vegetation area increased from 824.1 to 902.4 km^2^ during 1995–2015.

After the operation of TGD, the total vegetation area extended more quickly (0.08 km^2^/year vs. 6.47 km^2^/year before and after 2003). The decline in Grass area was also more rapid after the operation of TGD (-1.37 km^2^/year vs. -1.66 km^2^/year before and after 2003). The area of Reed and Forest increased rapidly after the operation of TGD (1.45 km^2^/year vs. 8.13 km^2^/year before and after 2003) and covered 35.5% of the total vegetation area.

### Changes in Vegetation Distribution Elevation

**FIGURE 3:**
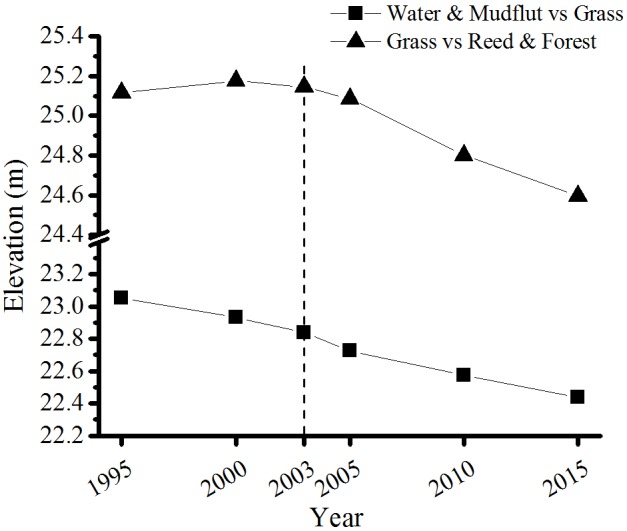
The lowest distribution elevation of both vegetation types during 1995–2015.

The lowest distribution elevation of Grass continually decreased from 23.06 m in 1995 to 22.44 m in 2015 (**Figure 3**). The lowest distribution elevation of Reed and Forest also showed a declining trend, slightly decreasing during 1995–2003, but quickly declined from 25.08 m in 2005 to 24.59 m in 2015. Over the 20-year period, the lowest distribution elevation had moved down by about 0.61 m for Grass and about 0.52 m for Reed and Forest. In both vegetation types, the decline of the lowest distribution elevations was faster after the operation of TGD. The decline rate of the lowest distribution elevation was 2.4 cm/year vs. 3.1 cm/year in the Grass before and after 2003, and -0.3 cm/year vs. 4.2 cm/year for Reed and Forest.

### Changes in Monthly Water Level and Submergence Duration

**FIGURE 4:**
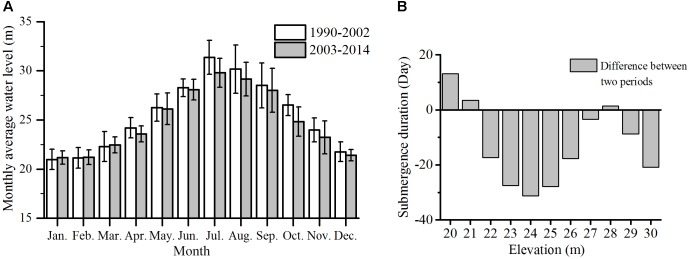
**(A)** Changes in average monthly water level and submergence duration between 1995–2002 and 2003–2014. **(B)** Negative and positive values indicate decrease and increase in duration, respectively, after the operation of Three Gorges Dam.

Compared to the average monthly water level during 1990–2002, water level was 0.08–0.20 m higher from January to March, but 0.14–1.69 m lower from April to December after the operation of TGD. In July, August, and October, average monthly water level decreased by more than 1 m after the operation of TGD (**Figure 4A**). As a result of water level variation, the average submergence duration during 1990–2002 and 2003–2014 also changed at different elevations (**Figure 4B**). After the operation of TGD, submergence duration increased at 20, 21, and 28 m elevations (1–13 days), but significantly decreased at 22–27 m and 29–30 m elevations (-3 to -31 days), especially at 23–25 m elevations (<-27 days).

### Submergence Duration at the Lowest Distribution Elevation of Vegetation Distribution

The 5-year submergence duration was significantly correlated with the lowest elevation distribution in both vegetation types (*R*^2^ = 0.64). The submergence duration was 233–257 days for Grass and 160–192 days for Reed and Forest during the six periods, respectively. The average submergence duration was 246 days for Grass and 177 days for Reed and Forest, respectively.

**FIGURE 5:**
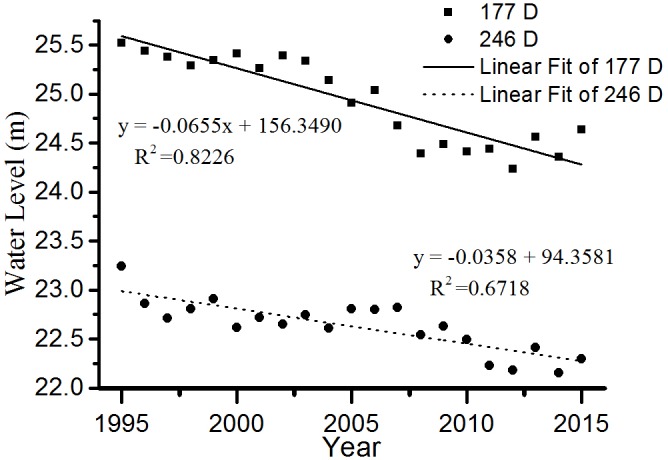
Changes of water level corresponding to submergence duration 177 and 246 days during 1995–2015.

Based on the average submergence durations of Grass and Reed and Forest, we can get the corresponding elevation in any year using the daily water level data. Linear regression analyses were then conducted to elucidate the change trend of distribution elevation in both vegetation types during 1995 – 2015 (**Figure 5**). Both linear regression equations were statistically significant (for Grass, *F* = 41.9, *P* < 0.01; for Reed and Forest, *F* = 93.7, *P* < 0.01), suggesting that the distribution elevations of both vegetation types were consistently decreased during 1995–2015.

## Discussion

During the past two decades, the eco-hydrological environments of the EDTL have considerably changed, particularly after the operation of TGD (Feng et al., 2013). The dam has been modified the probabilistic regularity of the flooding periods (Zhang et al., 2012; Yuan et al., 2015; Han et al., 2016). As a result of changed water regime, the area of Water and Mudflat decreased about 5.5% (78.2 km^2^) of EDTL in winter season due to the rapid expansion of vegetation. Vegetation distribution has shown a new pattern. As a response to the decreased water level, the vegetation at high elevations tended to expand rapidly to the central part of the lake. Moreover, the vegetation replacement process has also been accelerated, especially after the operation of TGD, which is consistent with the findings of another study (Hu et al., 2015) and parallel to what happened in another lake in the middle reaches of the Yangtze River, the Poyang Lake, where one vegetation type has been completely converted to another (Li et al., 2016).

**FIGURE 6:**
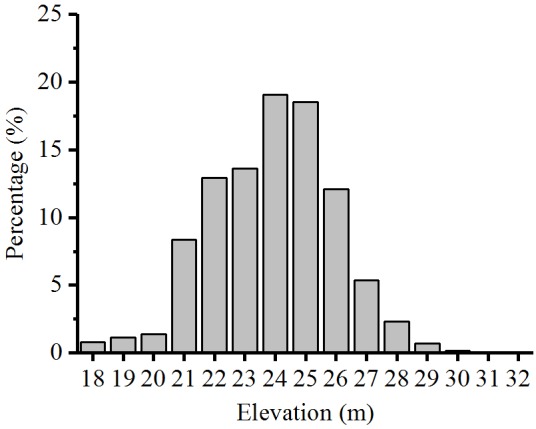
Elevation-area relationship in East Dongting Lake.

The total area and spatial distribution of the two vegetation types have also greatly changed. These results might be closely related to the topographic characteristics of EDTL (**Figure 6**). The land area at 21–27 m elevation covers 90% of total lake basin. In this study, the lowest distribution elevations for Grass and Reed and Forest were 23.06–22.45 m and 25.08–24.55 m, respectively. Therefore, rapid changes of water level at 22–25 m elevations (accounting for 64.14% of the total area) would lead to significant change in total vegetation area and vegetation distribution pattern.

The alteration of vegetation distribution is usually considered as an adaptive mechanism to the changed water level. After the operation of TGD, the decline of monthly average water level occurs earlier in either the annual flood season or dry season, consequently exposing the lake-basin to air for a longer time (Huang et al., 2014), which provides favorable condition for plant growth and development. As a result, the vegetation also had a longer time to recover and accumulate more energy after flooding, which might facilitate vegetation expansion in either Reed and Forest or Grass. Additionally, changed water regimes altered the lowest distribution elevation of each vegetation type. The dynamic balance between Grass and Reed and Forest was also changed by the lower water level, leading to the vegetation at lower elevation rapidly replaced by higher-elevation one. This may explain why Reed and Forest had a faster expansion rate compared to Grass (4.9 cm/year vs. 3.3 cm/year). The expansion area was mainly distributed between the elevations of 22–24 m, where used to be occupied by Water and Mudflat. It was clear that the changed water regimes at this elevation range might promote the expansion of Grass to Water and Mudflat. Therefore, Reed and Forest occupied part of the space of Grass and Grass occupied part of the space of Water and Mudflat.

Most wetland plants have formed adaptive strategy to the regular change in water regime through a long-term evolution process (Baldwin et al., 2001). According to the variance of water level at different elevations, the submergence duration clearly increased at 20–21 m elevations but decreased at 22–27 m elevations, particularly at 24 m. The lowest distribution elevation of Grass continually decreased from 23.06 m in 1995 to 22.44 m in 2015, and from 25.08 m in 2005 to 24.59 m in 2015 for Reed and Forest. In this study, average submergence duration was 246 days for Grass and 177 days for Reed and Forest, respectively. Although the lowest distribution elevations for Grass and Reed and Forest were continually decreased during 1995–2015, the average submergence duration corresponding to the lowest distribution elevation was relatively constant in either Grass or Reed and Forest. Therefore, submergence duration may be a good indicator for predicting the change of vegetation distribution.

The characteristic of individual plant species can also account for the change of vegetation distribution. The Grass, mainly Carex species (Cyperaceae), has found to be a strong tolerance to long-time submergence (Li et al., 2013). Carex has two growing seasons in Dongting Lake wetlands, and can grow immediately when the flooding is receded. In contrast, the Reed and Forest has a higher tolerance to drought but lower tolerance to flooding (Li et al., 2013). Along with the declining water level, the advantage of high flood tolerance for Grass was weakened and it was easily replaced by the Reed and Forest, which can grow more favorably in areas with low water level (Deng et al., 2014).

As a member of Ramsar convention, Dongting Lake contains approximately 1420 plant species, 114 fish species and 217 bird species and has been listed as one of the 200 global conservation priority eco-regions proposed by the WWF. The Grass-dependent animals and migratory birds may be impacted by the change of habitat structure, as a result of degeneration of Grass. Change of water regime also accelerated human disturbance, which might be a risk for the wetland ecosystem (Zhao and Fang, 2004). For example, black poplar (*Populus nigra*) was introduced to plant in the Dongting Lake for economic profitability (Li et al., 2014). These activities have accelerated the change of vegetation dynamic and vegetation distribution, although some *Populus* trees have been cut down by local government. Therefore, how to maintain the ecosystem stability and structural integrity in Dongting lake wetlands is an important problem, especially after the operation of TGD. Further model research on the relationship between vegetation distribution and water regime should be conducted to better predict the change trend of vegetation distribution, which could provide scientific guidance for vegetation restoration and wetland management in this lake.

## Author Contributions

J-YH analyzed the remote sensing data and wrote the manuscript. Y-HX designed the initial framework. YT provided a part of the water level data and the classification method. FL and Y-AZ contributed to revising the manuscript. The ideas of this paper were generated through discussions by all authors.

## Conflict of Interest Statement

The authors declare that the research was conducted in the absence of any commercial or financial relationships that could be construed as a potential conflict of interest.
